# The role of glucose metabolism and insulin resistance in cardiac remodelling induced by cigarette smoke exposure

**DOI:** 10.1111/jcmm.16053

**Published:** 2020-12-09

**Authors:** Paula Schmidt Azevedo, Bertha F. Polegato, Sergio Paiva, Nara Costa, Priscila Santos, Silmeia Bazan, Ana Angelica Henrique Fernandes, Alexandre Fabro, Vanessa Pires, Suzana E. Tanni, Filipe Leal Pereira, Angelo Lo, Leticia Grassi, Dijon Campos, Vickeline Androcioli, Leonardo Zornoff, Marcos Minicucci

**Affiliations:** ^1^ Department of Internal Medicine Botucatu Medical School São Paulo State University‐UNESP Botucatu Brazil; ^2^ Faculty of Nutrition UFG – Univ Federal de Goiás Goiânia Brazil; ^3^ Department of Chemical and Biological Sciences São Paulo State University‐UNESP Botucatu Brazil; ^4^ Department of Pathology and Legal Medicine Ribeirão Preto Medical School University of São Paulo Ribeirão Preto Brazil; ^5^ Experimental Research Unit – UNIPEX Botucatu Medical School São Paulo State University‐UNESP Botucatu Brazil

**Keywords:** cardiac remodelling, cigarette smoke, glucose metabolism, heart failure, ventricular remodelling

## Abstract

The aim of this study is to evaluate whether the alterations in glucose metabolism and insulin resistance are mechanisms presented in cardiac remodelling induced by the toxicity of cigarette smoke. Male Wistar rats were assigned to the control group (C; n = 12) and the cigarette smoke‐exposed group (exposed to cigarette smoke over 2 months) (CS; n = 12). Transthoracic echocardiography, blood pressure assessment, serum biochemical analyses for catecholamines and cotinine, energy metabolism enzymes activities assay; HOMA index (homeostatic model assessment); immunohistochemistry; and Western blot for proteins involved in energy metabolism were performed. The CS group presented concentric hypertrophy, systolic and diastolic dysfunction, and higher oxidative stress. It was observed changes in energy metabolism, characterized by a higher HOMA index, lower concentration of GLUT4 (glucose transporter 4) and lower 3‐hydroxyl‐CoA dehydrogenase activity, suggesting the presence of insulin resistance. Yet, the cardiac glycogen was depleted, phosphofructokinase (PFK) and lactate dehydrogenase (LDH) increased, with normal pyruvate dehydrogenase (PDH) activity. The activity of citrate synthase, mitochondrial complexes and ATP synthase (adenosine triphosphate synthase) decreased and the expression of Sirtuin 1 (SIRT1) increased. In conclusion, exposure to cigarette smoke induces cardiac remodelling and dysfunction. The mitochondrial dysfunction and heart damage induced by cigarette smoke exposure are associated with insulin resistance and glucose metabolism changes.

## 1 INTRODUCTION

A new, recently known form of smoking‐induced cardiac injury consists of the toxic effects cigarette smoke exerts directly on the myocardium, regardless of vascular factors. In previous studies, cigarette smoke‐induced different patterns of cardiac remodelling followed by myocardial dysfunction.^1^, ^2^, ^3^, ^4^, ^5^, ^6^, ^7^, ^8^

Considering the mechanisms involved in this phenomenon, the roles of glucose metabolism and insulin resistance in smokers' myocardium remain to be elucidated. Thus, this study's objective was to evaluate whether alterations in glucose metabolism participate in cardiac remodelling induced by the toxic effects of exposure to cigarette smoke.

## 2 MATERIALS AND METHODS

### 2.1 Study design

This research protocol was approved by the Animal Ethics Committee of Botucatu Medical School and was performed following the National Institute of Health's *Guide for the Care and Use of Laboratory Animals*.

Twenty‐four male Wistar rats, weighing between 200 and 250 g, were divided into two groups: the control group (not exposed to cigarette smoke) (C; n = 12) and the cigarette smoke‐exposed group (exposed to cigarette smoke over the 2 months) (CS; n = 12). The exposure to cigarette smoke for 2 months was performed according to the previously standardized method from our laboratory.^1^, ^5^

### 2.2 Caudal systolic pressure

The animals underwent caudal systolic pressure after 1 and 2 months of exposure to cigarette smoke as previously described.^2^

### 2.3 Echocardiographic analysis

All the rats were weighed and evaluated by a transthoracic echocardiographic examination (General Electric Medical Systems, Vivid S6). All measurements were performed by the same observer blinded to treatments and according to the American Society of Echocardiography/European Association of Echocardiography.^5^

### 2.4 Serum insulin, cotinine, catecholamine and glucose dosages

Insulin and cotinine were measured by the ELISA method. Plasma adrenaline, noradrenaline and dopamine analysis was performed by high‐performance liquid chromatography (HPLC). Glucose was dosed by enzymatic colorimetric method. The Homeostasis Model Assessment (HOMA) index was calculated using the equation (glucose (mmol/L) × insulin µU/mL)/22.5.^9^

### 2.5 Evaluation of oxidative stress, cardiac energy metabolism and GLUT4

Protein concentration, lipid hydroperoxide, antioxidant enzyme activity and energy metabolism activity were determined as previously described.^5^

The immunohistochemistry evaluation of GLUT4 was evaluated as previously described. To determine left ventricular myocardial glycogen, 20 mg samples were homogenized in 0.1 mol/L perchloric acid and centrifuged (1400 g/10 min). Glycogen concentration was calculated by the difference between final and initial glucose.^10^

### 2.6 Western blot

Cytoplasmic proteins AKT, phospho‐AKT, AMPK and SIRT‐1 were extracted from cardiac tissue using RIPA buffer.

### 2.7 Statistical analysis

The variables with normal distribution are presented as mean ± standard deviation and the variables with non‐normal distribution as median and 25th and 75th percentiles. The comparisons between the groups were made by the Student *t* test or Mann‐Whitney, according to the normality of variables distribution. Data analysis was performed using SigmaPlot software for Windows v12.0 (Systat Software Inc). The level of significance was 5%.

## 3 RESULTS

**FIGURE 1:**
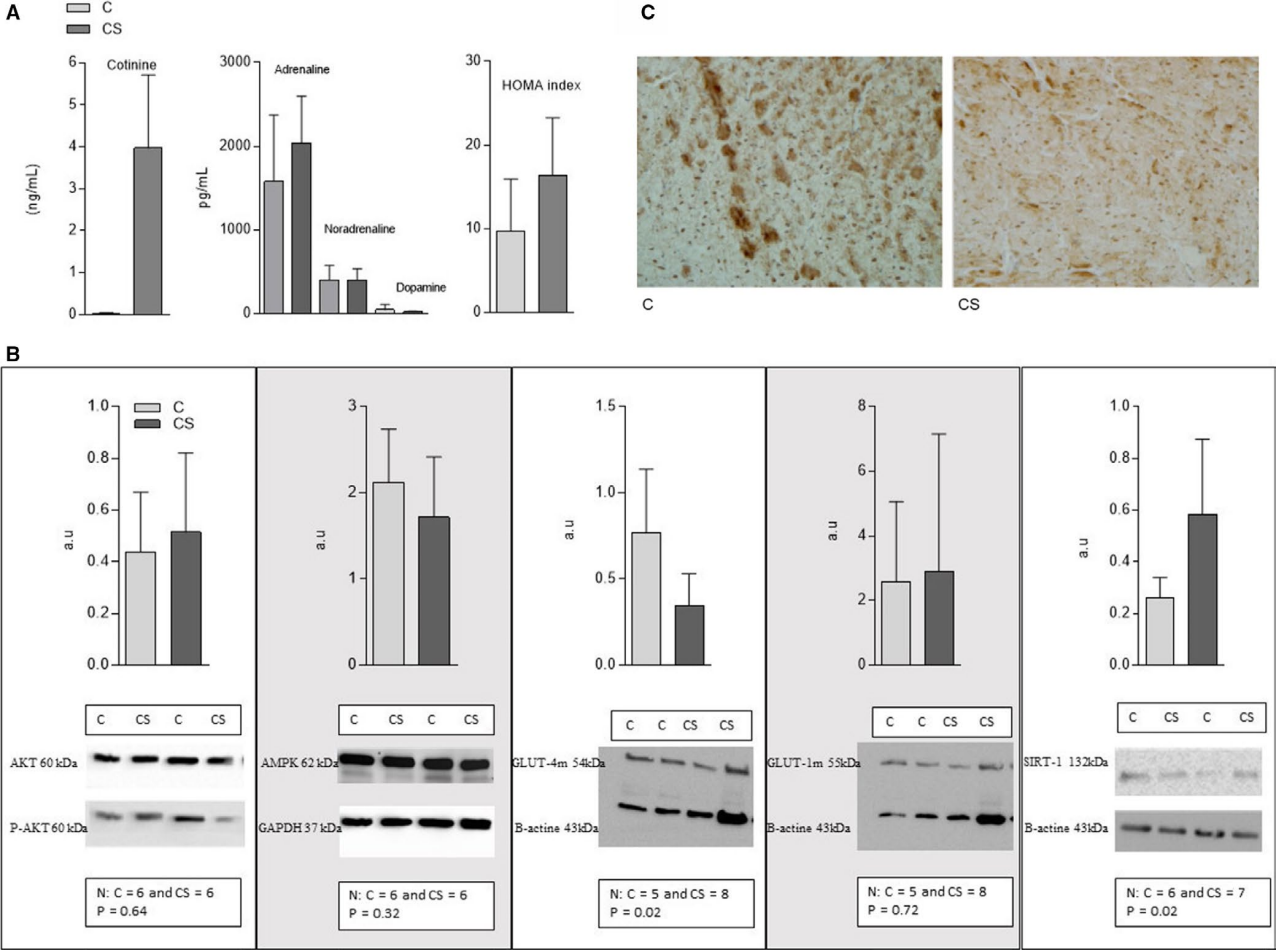
A, Cotinine and catecholamine serum concentrations and HOMA index of rats exposed and not exposed to cigarette smoke. C: control group, not exposed to cigarette smoke; CS: group exposed to cigarette smoke. N = number of animals; *P* = 5% significance level; HOMA index [glycemia (mmol) × insulin (m UI)/22.5]. Cotinine: N = 12 per group, *P* < .001; Catecholamines: N = 10 per group, adrenaline *P* = .16, noradrenaline *P* = .95, dopamine *P* = .11; HOMA Index: N = 12 per group, *P* = .02. B, Expression of proteins involved in cardiac energy metabolism in animals exposed or not to cigarette smoke. C = control group—not exposed to cigarette smoke; CS: group exposed to cigarette smoke N = number of animals in each group; *P* < .05 significance level. AKTt: Total Protein Kinase B; AKTf: phosphorylated protein kinase B; AMPK: adenosine monophosphate‐activated protein kinase; SIRT1: silent information regulator—sirtuin 1; GLUT4m: membrane glucose transporter 4; GLUT1m: membrane glucose transporter 1. C, Expression of glucose transporter 4 (GLUT4) analysed by immunohistochemistry. C = control group—not exposed to cigarette smoke; CS = group exposed to cigarette smoke

The serum levels of cotinine, catecholamines and the HOMA index are shown in Figure 1.

Systolic blood pressure was similar in both groups (C = 197 ± 18.2; CS = 191 ± 21.8; *P* = .48). CS group showed an increase in left atrium, relative wall thickness and left ventricle mass index, whereas left ventricle diastolic diameter decreased. The structural findings were accompanied by diastolic dysfunction observed by higher E wave deceleration time and systolic dysfunction evidenced by lower ejection fraction (Table 1).

**TABLE 1:** Echocardiography, cardiac energy metabolism and oxidative stress of rats exposed and not exposed to cigarette smoke

	C (N = 12)	CS (N = 12)	*P*
LA area (mm^2^)	16.9 ± 2.48	24.2 ± 1.42	<.001
LVDD (mm)	7.08 ± 0.55	6.64 ± 0.36	.03
LVSD (mm)	3.14 ± 0.39	3.13 ± 0.25	.95
PWT (mm)	1.49 (1.45‐1.53)	1.82 (1.79‐2.018)	<.001
RWT	0.42 ± 0.04	0.55 ± 0.06	<.001
LVMI (kg/m^2^)	1.47 ± 0.28	1.83 ± 0.19	.001
EDT (s)	44.0 (39.0‐45.0)	55.5 (51.0‐58.0)	.004
HR (bpm)	274.0 ± 25.0	255 ± 15.0	.04
EF %	0.91 ± 0.02	0.89 ± 0.02	.04
PFK (nmol/g)	138.34 ± 24.18	198.72 ± 31.57	.003
LDH (µmol/mg)	121.90 ± 31.33	167.84 ± 27.43	.017
PDH (nmol/g)	208.64 ± 18.83	211.14 ± 27.70	.863
CS (µmol/mg)	39.04 ± 2.32	25.53 ± 5.36	<.001
3‐OH‐acyl CoA‐DH (nmol/mg)	41.57 ± 4.42	14.55 ± 2.78	<.001
Complex I (nmol/mg)	5.32 ± 0.48	2.75 ± 0.62	<.001
Complex II (nmol/mg)	5.32 ± 0.93	2.48 ± 0.78	<.001
ATP synthase (nmol/mg)	57.91 ± 7.24	21.18 ± 4.84	<.001
Glycogen (mg/g)	40.8 ± 5.23	17.5 ± 1.40	<.001
HPX (nmol/g)	205.0 ± 6.9	450.0 ± 23.7	<.001
Catalase (µmol/g)	44.9 ± 3.1	37.1 ± 2.0	.04
SOD (nmol/g)	7.00 ± 0.42	6.70 ± 0.32	.59

Abbreviations: LA: left atrium; W: LVDD: left ventricular diastolic diameter; LVSD: left ventricular systolic diameter; PWT: posterior wall thickness; RWT: relative thickness (2PWT/LVDD); LVMI: left ventricular mass index; EDT: E wave deceleration time; HR: heart rate in bpm (beats per minute); EF: ejection fraction. PFK: phosphofructokinase; PDH: pyruvate dehydrogenase; LDH: lactate dehydrogenase; CS: citrate synthase; 3‐OHAcylCoA‐DH: 3‐hydroxy‐acyl‐coenzyme A dehydrogenase; ATP: adenosine triphosphate; HPX: lipid hydroperoxide; SOD: superoxide dismutase. C = control group, not exposed to cigarette smoke; CS: group exposed to cigarette smoke; N = number of animals (echocardiogram = 12, cardiac energy metabolism and oxidative stress = 8); *P* < .05 significance level.

The CS altered cardiac energy metabolism and generated oxidative damage. Cardiac glycogen was lower in the CS group. Moreover, HP formation also increased, accompanied by lower catalase activity in the CS group (Table 1).

The proteins responsible for regulation of cardiac energetic metabolism have also changed, as shown in Figure 1. In the CS group, the membrane GLUT4 decreased and SIRT1 increased. The other proteins studied as AKTf/AKTt, AMPK and membrane GLUT‐1 were similar in both groups. The qualitative evaluation of GLUT4 via immunohistochemistry showed a pattern of reduced fraction area of this protein in the CS group.

## 4 DISCUSSION

The remodelling process induced by exposition to cigarette smoke is it is well characterized, both in experimental and clinical studies.^1^, ^2^, ^3^, ^4^, ^5^, ^6^, ^7^, ^8^ However, the mechanisms that underlie smoke cardiomyopathy are not well understood.

Therefore, we hypothesized that insulin resistance is central to this process. Our explanation for cigarette smoke‐induced insulin resistance is based, in part, on the role of nicotine‐induced adrenergic activity in stimulating lipolysis and the release of fatty‐free acid (FFA) into the blood circulation.^11^ FFAs are uptaken by myocytes but cannot be fully metabolized, instead accumulating in the cell as triglycerides or ceramides. This process, termed lipotoxicity, is key to the development of insulin resistance and was previously described in cigarette smoke settings. Indeed, in this study, we observed the presence of insulin resistance, marked by an increase in HOMA index, lower membrane GLUT‐4 concentration, and lower GLUT‐4 labelling in quantitative and qualitative immunohistochemical analysis in animals exposed to cigarette smoke.^11^ Moreover, in this study, FA beta‐oxidation evaluated by 3‐hydroxy‐acyl Coenzyme A dehydrogenase activity was reduced. Thus, excess FA uptake may have accumulated in myocytes, thus contributing to insulin resistance.^4^

Another factor that reinforces our hypothesis is that a previous study has already shown that beta‐blockers attenuate cigarette smoke‐induced cardiac damage, highlighting the role of adrenergic pathway and cardiac remodelling.^12^

The following steps of this investigation were to identify how energy substrates were metabolized in the cigarette smoke exposure scenario, considering two highly important energy generation pathways: glycolysis and mitochondrial oxidative phosphorylation.^13^ One important finding was that the cardiac glycogen in animals exposed to cigarette smoke was reduced and may have been consumed because glucose uptake was impaired. Glycogen is a key source of energy in stressful situations where glycogenolysis is the main source of glucose generation.^14^

Regarding the glycolysis pathway, despite the probable impairment of glucose uptake by GLUT4, our data showed that the activity of the enzyme PFK and LDH was increased in the group exposed to cigarette smoke but that the PDH did not change. PFK‐1 is the key enzyme in the glycolytic pathway, stimulated by insulin and the adrenergic pathway.^13^, ^14^ However, in an experimental model in rats with type 1 diabetes, maintenance of the action of the enzyme PFK‐1 and increase of intermediate products of the glycolytic pathway were observed, probably from some glucose uptake, regardless of insulin and adrenergic action.^14^ Additionally, the increase in LDH in the absence of PDH suggests that the pyruvate formed is being converted to lactic acid. This probably contributed to certain tissue acidosis and consequently aggravated myocardial dysfunction.^15^

Finally, our data confirmed the participation of oxidative stress and mitochondrial changes in smoking‐induced remodelling. Indeed, the present study evidenced lipid membrane damage, detectable by higher levels of lipid hydroperoxide concentration and lower activity of catalase, an antioxidant enzyme. Likewise, we observed mitochondrial dysfunction and ineffective oxidative phosphorylation, which promote stimulation of anaerobic glycolysis, which further generates lactic acid.^15^ In addition, we hypothesized that mitochondrial dysfunction, indirectly seen in the reduction of citrate synthase activity, mitochondrial complexes and ATP synthase, increased SIRT1 expression to remove damaged organelles by mitophagy. Another possibility is that the higher expression of SIRT1 contributed to hypertrophy as a causal factor.

In conclusion, exposure to cigarette smoke induces cardiac remodelling and dysfunction. The mitochondrial dysfunction and heart damage induced by cigarette smoke exposure are associated with insulin resistance and glucose metabolism changes.

## CONFLICT OF INTEREST

No potential conflict of interest was reported by the authors.

## AUTHOR CONTRIBUTION

**Paula Schmidt Azevedo:** Conceptualization (lead); Data curation (equal); Formal analysis (equal); Funding acquisition (lead); Investigation (equal); Project administration (equal); Writing‐original draft (lead); Writing‐review & editing (lead). **Bertha F Polegato:** Formal analysis (lead); Investigation (lead); Writing‐original draft (lead). **Sergio Paiva:** Formal analysis (equal); Supervision (lead); Writing‐review & editing (equal). **Nara Costa:** Data curation (equal); Investigation (equal); Writing‐original draft (equal); Writing‐review & editing (equal). **Priscila Santos:** Data curation (equal); Investigation (equal); Writing‐original draft (equal); Writing‐review & editing (equal). **Silmeia Bazan:** Investigation (equal); Methodology (equal); Writing‐review & editing (equal). **Ana Angelica Henrique Fernandes:** Investigation (equal); Methodology (equal). **Alexandre Fabro:** Investigation (equal); Methodology (equal); Writing‐review & editing (equal). **Vanessa Pires:** Formal analysis (equal); Investigation (equal); Methodology (equal). **Suzana E Tanni:** Data curation (equal); Formal analysis (equal); Investigation (equal); Supervision (equal). **Filipe Leal‐Pereira:** Investigation (equal); Methodology (equal). **Angelo Lo:** Investigation (equal); Methodology (equal). **Leticia Grassi:** Investigation (equal); Methodology (equal). **Dijon Campos:** Investigation (equal); Methodology (equal). **Vickeline Androcioli:** Investigation (equal); Methodology (equal). **Leonardo Zornoff:** Formal analysis (equal); Supervision (lead); Writing‐review & editing (supporting). **Marcos Minicucci:** Conceptualization (equal); Formal analysis (equal); Project administration (equal); Supervision (lead); Writing‐review & editing (lead).
